# Siloxene Nanosheets and Their Hybrid Gel Glasses for Broad-Band Optical Limiting

**DOI:** 10.3390/molecules28052143

**Published:** 2023-02-24

**Authors:** Xugui Lv, Nan Li, Yunfei Li, Qingyu Ma, Zheng Xie, Shuyun Zhou

**Affiliations:** 1 School of Materials Science and Engineering, University of Jinan, Jinan 250022, China; 2 Key Laboratory of Photochemical Conversion and Optoelectronic Materials, Technical Institute of Physics and Chemistry, Chinese Academy of Sciences, Beijing 100190, China

**Keywords:** siloxene nanosheets, nonlinear absorption, hybrid gel glasses, broad-band, optical limiting

## Abstract

With the development of laser technology, the research of novel laser protection materials is of great significance. In this work, dispersible siloxene nanosheets (SiNSs) with a thickness of about 1.5 nm are prepared by the top-down topological reaction method. Based on the Z-scan and optical limiting testing under the visible-near IR ranges nanosecond laser, the broad-band nonlinear optical properties of the SiNSs and their hybrid gel glasses are investigated. The results show that the SiNSs have outstanding nonlinear optical properties. Meanwhile, the SiNSs hybrid gel glasses also exhibit high transmittance and excellent optical limiting capabilities. It demonstrates that SiNSs are promising materials for broad-band nonlinear optical limiting and even have potential applications in optoelectronics.

## 1. Introduction

At present, due to the development of high-energy lasers and related technologies, the applications of laser technology in basic scientific research, and biomedical and defense industries are growing rapidly [1,2]. However, high-energy lasers are prone to causing serious damage to the human eyes, optical sensors, etc., which has greatly stimulated the development of laser protective materials. Currently, widely-used laser protection includes absorption filters and reflective filters based on linear optics, as well as nonlinear optical limiting materials. The nonlinear optical limiting capabilities of a material are based on its nonlinear optical effects and are expected to meet the requirements for efficient laser protection, including broad-band optical limiting, high linear transparency, and low nonlinear transmittance under a high energy laser [3]. Thus far, research on optical limiting materials has gradually come to involve two-dimensional nanomaterials and organic materials, represented by graphene, over the initial inorganic semiconductor materials [2].

As the two-dimensional form of graphite, graphene has extremely outstanding properties, and a large number of two-dimensional materials have attracted peoples’ attention due to their potential properties and applications [4,5]. As one of the most abundant elements in the earth’s crust, silicon (Si) has become an essential and ubiquitous chemical element in modern life and industry because of its non-toxic, biocompatible, and environmentally-friendly properties. Silicene is two-dimensional crystals consisting of a six-membered ring, similar to graphene, with C atoms replaced by Si atoms in its planar framework [6]. Although C and Si belong to the same main-group, graphene and silicene are still structurally different. Due to the larger size of the single atom compared to carbon atoms, the silicon atom has better stability in sp^3^ hybridization [7]. As consequence, the C atoms in graphene can be arranged into a perfect planar structure, while the Si atoms in silicene are more inclined to hybridize sp^2^ and sp^3^, forming a periodic topology [8,9,10].

Siloxene is a partially oxidized, two-dimensional Si with various oxidation functional groups on its surface. Siloxene has distinctive physical and chemical properties due to its large specific surface area and unique quantum confinement effects [6,11,12,13,14]. Research on siloxenes has now progressed to a variety of silicon structures [15,16,17,18,19]. Depending on the degree of oxidation of the siloxene, as well as the surface groups and skeletal structure, three types can be distinguished: Weiss type siloxene, in which the Si_6_ plane is connected with -H and -OH groups, the Si (111) plane remains intact, with almost no bridging oxygen interspersed, -H or -OH ligands exist in the form of suspended bonds around the Si atoms, and the siloxene is metastable; Chain siloxene, this type of siloxene nanosheets (SiNSs) has -H ligands, and O atoms are interspersed in chains in the Si_6_ plane in the form of bridges, and this kind of structure also has lower energy and is relatively stable; Kautsky type siloxene, in which the Si_6_ is connected with bridging oxygen and terminated with an -H ligand, in which each silicon atom is bonded to the other two silicon atoms and an oxygen atom, which can exist more stably. According to previous reports, siloxene has broad application prospects in the fields of energy storage [18,20,21], electrochemistry [18,20,21], optoelectronics [22], and supercapacitors [23,24].

In general, the majority of two-dimensional materials have excellent nonlinear optical properties and are widely studied in photonics and optoelectronics [25]. Graphene, a pioneer of two-dimensional materials, has been widely studied and applied due to its excellent nonlinear optical properties [26,27], and provides solid foundations for the studies of other two-dimensional materials. The nonlinear optical properties of silicene nanosheets and their derivatives have been reported. Dodecyl-functionalized silicane has been prepared using hydrosilylation reactions, revealing a comparable or even stronger nonlinear optical response than graphene [28,29,30]. By liquid phase exfoliation, the crystalline silicon is exfoliated into silicene nanosheets, which convert the saturated absorption to the reverse saturated absorption as the power density increases [31]. As graphite-like bulk silicene or silicon-based materials do not exist in nature, it is not possible to obtain silicene nanosheets by simple mechanical exfoliation in the same way as graphene. However, calcium silicide is a stable binary Zintl compound, in which Si atoms construct a hexagonal graphene-like structure in the form of a negative oxidation state. Therefore, structurally complete, few-layer, and two-dimensional Si can be obtained by de-Ca treatment of calcium silicide.

**Scheme 1:**
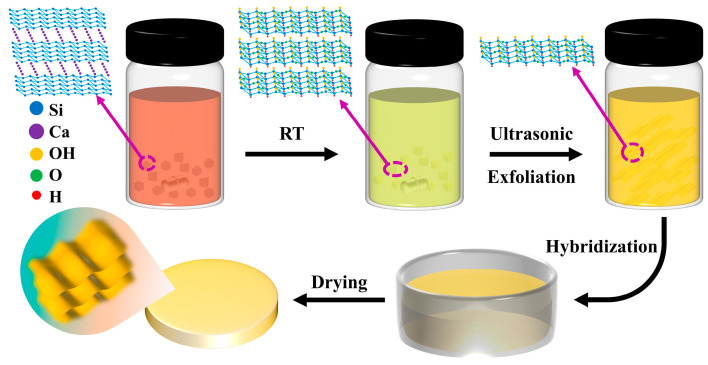
Schematic diagram of the preparation of siloxene nanosheets and their hybrid gel glasses.

Herein, we used calcium silicide (CaSi_2_) as the raw material and ferric chloride (FeCl_3_) as the oxidizing agent to prepare few-layer SiNSs using a topochemical reaction method in ethyl acetate (as shown in Scheme 1) [32]. The calcium is dissolved in the solvent as calcium chloride and the silicon layer is converted to SiNSs by the effect of iron ions and the solvent. The SiNSs are dispersible, and their thickness is about 1.5 nm. The hybrid gel glasses of SiNSs exhibit strong optical limiting behavior. SiNSs have proved to be a potential nonlinear optical material and their hybrid gel glasses are high-transmittance glass devices that can be used for optical limiting.

## 2. Results and Discussion

### 2.1. Morphological and Elemental Characterization of Siloxene Nanosheets

**Figure 1:**
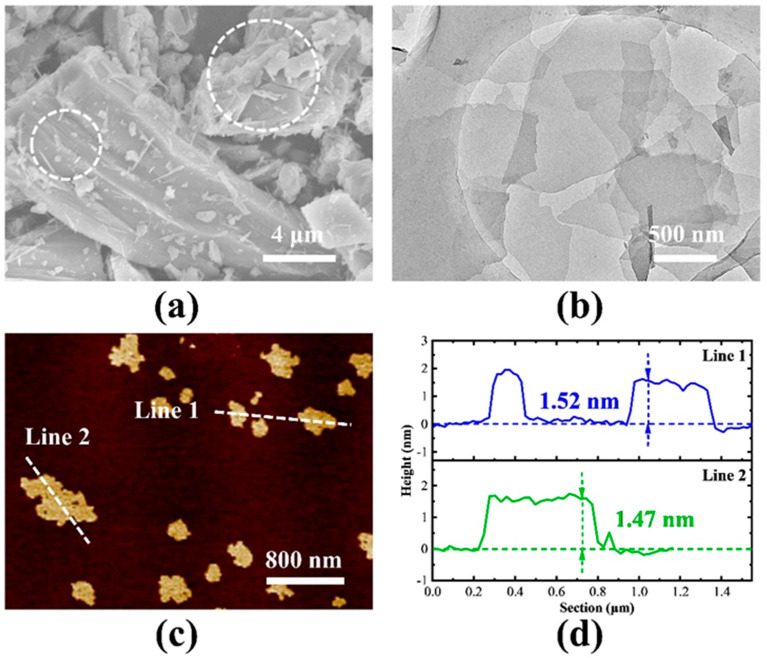
Microscopic morphology of CaSi_2_ powders and siloxene nanosheets. SEM image of CaSi_2_ (**a**), TEM (**b**), AFM image (**c**), and corresponding heights (**d**) of siloxene nanosheets.

Figure 1a shows the representative SEM image of CaSi_2_ powders. The CaSi_2_ powders before the experimental treatment were mainly in the form of lumpy particles. The CaSi_2_ crystal is relatively complete and there is obvious stratification at the edges, as seen in the white dashed circle. After oxidation decalcification, the ethanol dispersion of the nanosheets was subjected to ultrasonic exfoliation. Ultrasound was used to counter the Van der Waals forces between the layers of the nanosheets and to weaken the interlayer interactions. Figure 1b shows representative TEM image of SiNSs. The SiNSs which are dispersed show an irregular superposition distribution, have a relatively low contrast, and are almost transparent, indicating that they have been exfoliated completely. The precise thickness of SiNSs was measured by AFM. SiNSs were dispersed in ethanol, and then added dropwise to the mica sheet. As shown in Figure 1c,d, the thickness of multiple differently sized samples was measured, and the thickness of the nanosheets was within 2 nm, indicating that the sample was composed of few-layer SiNSs [11].

**Figure 2:**
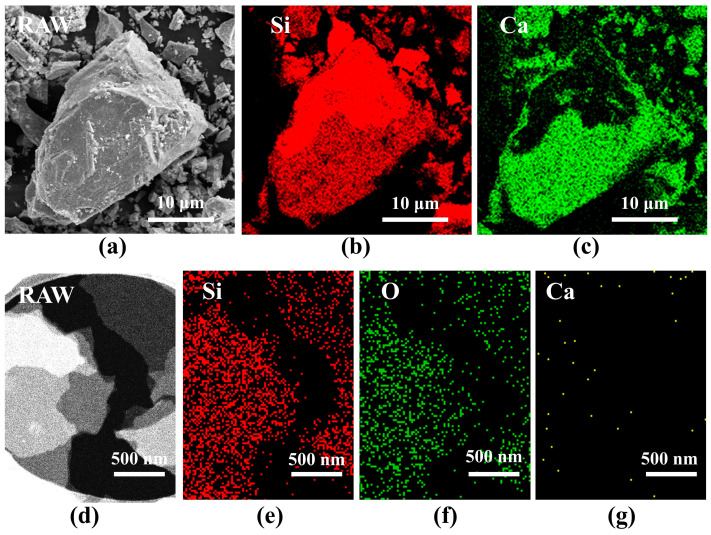
The element distribution of CaSi_2_ and siloxene nanosheets. (**a**–**c**) EDS images of CaSi_2_, (**d**–**g**) TEM mapping images of siloxene nanosheets.

In order to observe the elemental composition of CaSi_2_ powders and SiNSs, SEM Energy Dispersive Spectroscopy and TEM mapping were carried out for CaSi_2_ powders and SiNSs, respectively. Figure 2a–c shows the element distribution of CaSi_2_, which is dominated by Si and Ca elements, and the uneven distribution of elements is mainly due to the fact that the mapping test can only scan the surface layer. Figure 2d–g shows the element distribution of the SiNSs. The nanosheets are mainly composed of Si and O elements, and were almost free of Ca elements, indicating that the oxidative decalcification process was successfully carried out. Table 1 shows the elemental content of CaSi_2_ powders and SiNSs. The atomic proportion of Ca element in the nanosheets was 0.18%, which is almost negligible.

### 2.2. Structure and Elemental Characterization of Siloxene Nanosheets

**Figure 3:**
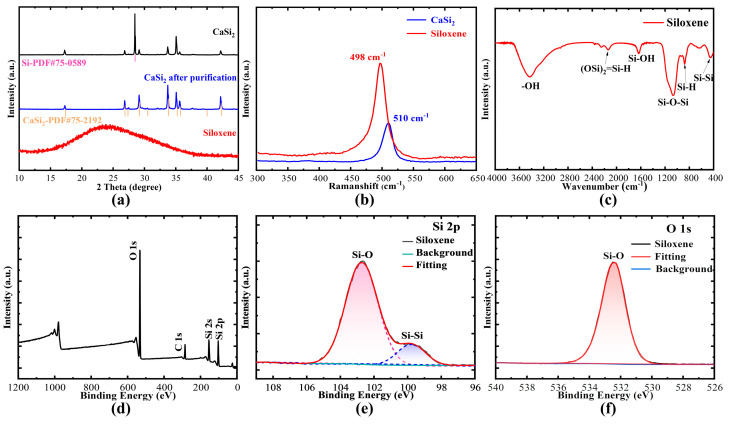
Structure and elemental characterization of siloxene nanosheets. (**a**) XRD patterns of CaSi_2_ powders, purified CaSi_2_ powders, and siloxene nanosheets; (**b**) Raman spectra of CaSi_2_ powders and siloxene nanosheets; (**c**) FT-IR spectrum; (**d**) X-ray photoelectron spectra for full range scan; (**e**) fitting curves of the Si 2p core level; (**f**) fitting curves of the O 1s core level of siloxene nanosheets.

The XRD patterns are shown in Figure 3a. The preparation process of commercial CaSi_2_ powders led to excessive crystalline silicon impurities, showing a typical crystalline silicon structure based on a peak of 28.5° (111). Therefore, commercial CaSi_2_ powders must be purified before use. The XRD pattern of the CaSi_2_ powders purified by an aqueous NaOH solution is shown in the blue curve of Figure 3a. The CaSi_2_ crystal remained intact, while the diffraction peaks of the crystalline silicon completely disappeared. SiNSs were prepared using purified CaSi_2_ powders. In the CaSi_2_ crystal, Si atoms are arranged in a long range. In the process of decalcification, the order of some Si atoms remain orderly. However, the two-dimensional Si plane has high surface energy. A large number of oxygen elements and oxygen-containing functional groups in the solvent react with the Si plane or enter the Si plane, and a large number of ordered Si planes are oxidized into disordered SiNSs [32]. The SiNSs’ powders exhibit an amorphous bulging peak state of 15–35°, as shown in the red curve of Figure 3a. In order to reflect the structure and quality of SiNSs, Raman spectra were used to detect the raw material CaSi_2_ powders and SiNSs, as shown in Figure 3b. CaSi_2_ powders have a sharp peak at 510 cm^−1^, and the exfoliated SiNSs have a sharp peak at 498 cm^−1^. The exfoliated nanosheets are obviously offset to a lower frequency [31,33], which indicates that the nanosheets were effectively exfoliated. The bonding nature of the functional groups present in SiNSs was examined using FT-IR, as shown in Figure 3c. The FT-IR spectrum of SiNSs shows the presence of broad vibration peaks at 2137, 1637, 1070, 876, and 460 cm^−1^, which corresponds to the vibration of ν(OSi_2_≡Si−H), ν(Si−OH), ν(Si−O−Si), ν(Si−H), and ν(Si−Si), indicating that SiNSs have a Kautsky type siloxene structure [21,32]. The broad peak observed at 3400 cm^−1^ corresponds to the hydroxyl group on the surface of the SiNSs. The main elemental composition of SiNSs and the chemical composition and valence state were determined by XPS measurements, as shown in Figure 3d,f. In the survey, it can be clearly seen that the main peaks are Si 2p, Si 2s, C 1s, and O 1s, which correspond to the center positions of about 102.63, 153.54, 284.83, and 532.21 eV, respectively. In order to better understand the oxidation state of Si elements in SiNSs, the Si 2p peak was fitted in Figure 3e. The Si 2p state energy spectrum can be mainly decomposed into two components: Si-Si bond and Si-O bond [21], for which the corresponding center positions are 99.78 and 102.7 eV, and the O 1s peak is fitted, as shown in Figure 3f; the fitting center peak position is 532.3 eV, corresponding to the peak position of the Si-O bond, which conforms to the Kautsky type SiNSs structure. The proportion of elements in the SiNSs is shown in Table 2. Due to the inevitable contact with oxygen during the preparation and testing of SiNSs, some Si elements will form amorphous SiO_2_, resulting in a higher content of O elements.

### 2.3. The Nonlinear Optical Properties of Siloxene Nanosheets

**Figure 4:**
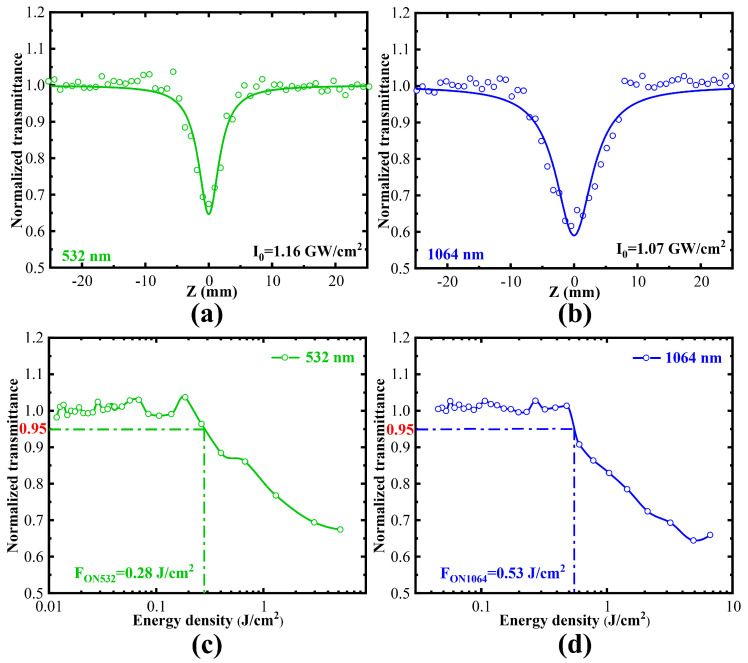
Nonlinear optical properties of siloxene nanosheet dispersions. (**a**,**b**) Z-scan results at 532 nm and 1064 nm; (**c**,**d**) Normalized transmittance and energy density curves at 532 nm and 1064 nm.

Figure 4a,b shows nonlinear optical curves of the SiNSs obtained by the nanosecond Z-scan system. As seen in Figure 4, the normalized transmittance decreases with the increase in power density, and the entire opening curves are in the shape of a downward trough when the energy reaches the nonlinear absorption threshold, showing a typical nonlinear absorption phenomenon. The normalized transmittance of the left and right shoulders in Figure 4a,b is symmetrical and equal, indicating that the sample did not experience irreversible damage caused by the laser heating. The normalized transmittance reaches the minimum value when the sample is at the focusing position (Z = 0). The optical modulation depth is 35% and the energy is 30 µJ under 532 nm laser pulse. Under 1064 nm, the optical limiting modulates the depth by 40%, and the energy is 160 µJ. The transmittance of the solvent without samples remains at the initial value (the normalized transmittance is 1). Figure 4c,d shows the curves of normalized transmittance and energy density converted from the curves in Figure 4a,b. Both normalized transmittance versus energy density curves can be divided into two phases, the linear absorption phase and the nonlinear absorption phase. When the laser energy density is small, the sample is in the linear absorption stage and the normalized transmittance of the sample is in a stable state; when the laser energy density increases to a certain threshold, the nonlinear optical properties of the sample are activated, and the normalized transmittance of the sample decreases continuously. After z_0_, the normalized transmittance of the sample gradually rises. In the nonlinear optical behavior, the corresponding energy density when the normalized transmittance is reduced to 95% is the onset limiting threshold (F_ON_) of the sample. The F_ON_ of SiNSs ethanol dispersion is 0.28 J/cm^2^ and 0.53 J/cm^2^ under 532 nm and 1064 nm laser pulses, respectively. Different from the previous report that SiNSs do not have saturation absorption [31], this resultt demonstrates that SiNSs have typical nonlinear optical limiting properties and have the potential to prepare laser protection devices.

The linear transmittance (T_linear_) of the samples at 532 nm and 1064 nm was 33% and 52%, respectively. Using the formula T_abs_ = exp(−α_0_L), substituting T_abs_ = T/T_0_ (T/T_0_, T is the transmittance of SiNSs ethanol dispersion + quartz cuvette, T_0_ is the transmittance of absolute ethanol + quartz cuvette) and when L is 0.1 cm, the linear absorption coefficient α_0_ at 532 nm and 1064 nm is 11.09 cm^−1^ and 6.54 cm^−1^. By fitting reverse saturation absorption data, the effective nonlinear absorption coefficient β_eff_ can be derived:

(1) $$T\left(z\right)\;\approx \;1-\frac{\beta_{\mathrm{eff}}I_{0}L_{\mathrm{eff}}}{2\sqrt{2}(1+\frac{z^{2}}{z_{0}{}^{2}})}$$

where I_0_ is the peak energy density at the focus (Z = 0), L_eff_ is effective length and can be derived from the formula: L_eff_ = [1 − exp(−α_0_L)], where z and z_0_ are the position of the sample on the *Z*-axis and the laser Rayleigh length, respectively. According to Equation (1), the fitting results are shown in Figure 4a,b. The effective nonlinear absorption coefficient β_eff_ at 532 nm and 1064 nm is 14.32 cm/GW and 14.80 cm/GW. The imaginary part of the third-order nonlinear susceptibility Imχ^(3)^ is derived from Equation (2), where c is the vacuum speed of light, n_0_ is the linear refractive index, and ω is the angular frequency of the excited light. The quality factor (FOM) of the third-order optical nonlinearity is expressed as FOM = |Imχ^(3)^/α_0_|. The results have been calculated and show that the Imχ^(3)^ and FOM of the SiNSs excited at 532 nm and 1064 nm are 6.09 × 10^−11^ esu and 5.47 × 10^−14^ m^4^/sW, 12.54 × 10^−11^ esu and 1.92 × 10^−13^ m^4^/sW.

(2) Imχ(3)=c2n02240π2ωβeff

### 2.4. The Nonlinear Optical Properties of Siloxene Nanosheets Hybrid Gel Glasses

**Figure 5:**
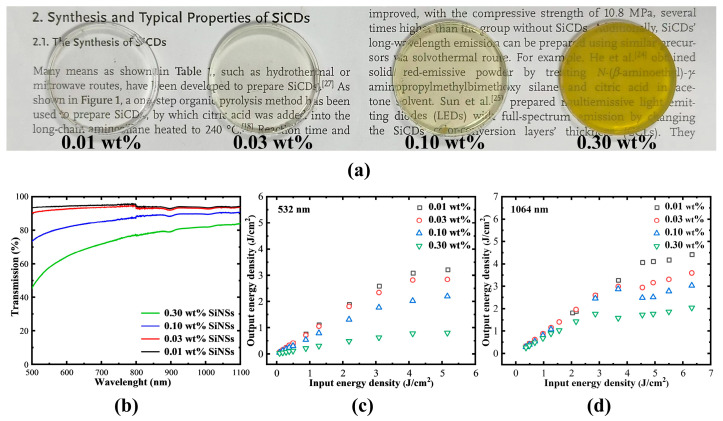
Nonlinear optical properties of siloxene nanosheet hybrid gel glasses. (**a**) Photographs (background text content [34]) and (**b**) visible-near IR spectra of 0.01, 0.03, 0.10, and 0.30 wt% siloxene nanosheets hybrid gel glasses; (**c**,**d**) optical limiting responses of siloxene nanosheet hybrid gel glasses under 532 nm and 1064 nm.

The Si-OH bonds on the surface of the nanosheets (containing Si-OH from the SiNSs themselves and Si-OH formed after hydrolysis of Si-H) are able to form Si-O-Si bonds by dehydration condensation with Si-OH in the gel formed after hydrolysis of the silane groups. After chemical hybridization, SiNSs can be perfectly combined with gel glass. Therefore, they were hybridized into gel glasses at different concentrations, as shown in Figure 5a. When the hybrid concentration reaches 0.3 wt%, SiNSs still do not agglomerate in their hybrid gel glasses. The prepared SiNSs hybrid gel glasses (SiNS-glasses) have a smooth surface and no cracks. As shown in Figure 5b, SiNS-glasses at different concentrations all have high transmittance at wavelengths in the visible-near IR ranges (the linear transmittances of hybrid glasses are >54%), which means that they have an application value. Optical limiting tests of gel glass with different concentrations were performed using a 532 nm and 1064 nm laser system. The test results are shown in Figure 5c,d. All SiNS-glasses with different hybrid concentrations showed exciting optical limiting signals. F_ON_ (the onset limiting threshold) is an important indicator to evaluate the nonlinear optical efficiency of materials or devices. The F_ON_ of the hybrid gel glass decreases with increasing doping of SiNSs. This demonstrates that the nonlinear optical efficiency of hybrid gel glasses increases significantly with increasing doping of the nanosheets. R_OL_ is the ratio of the output energy to the input energy at the maximum test energy in the optical limiting test. Comparing the difference value between T_linear_ and R_OL_, we can obtain a brief idea of the optical limiting capacity of the test sample. The gel glass with a doping concentration of 0.3 wt% has a stronger nonlinear optical property than gel glasses with other doping concentrations. SiNS-glasses with a hybrid concentration of 0.3 wt% exhibit the best optical limiting ability, indicating that the optical limiting ability of the gel glasses can be adjusted by changing different hybrid concentrations (Table 3). A comparison of the performance of SiNSs dispersions and hybridized gel glasses with common laser limiting materials is shown in Table 4. SiNSs have a relatively small F_ON_ both in anhydrous ethanol dispersions and in heterogeneous gel glasses, which can indicate that they are a good-performing nonlinear optical material. Moreover, the optical limiting effect of SiNS-glasses was observed from the visible-near IR ranges, demonstrating that the solid glasses prepared in this work are broad-band optical limiting devices.

## 3. Materials and Methods

### 3.1. Material Preparation

Calcium silicate (CaSi_2_, Gelest (Morrisville, PA, USA), technical grade), ferric chloride (FeCl_3_, Macklin (Shanghai, China), AR 99%), 36–38% hydrochloric acid (HCl (aq), Beijing Tongguang Fine Chemical Company (Beijing, China), AR), sodium hydroxide (NaOH, Macklin, AR 95%), ethanol (Peking Ragent (Beijing, China), AR 99.7%), ethyl acetate (Peking Ragent, AR 99.5%), deionized water, methyltriethoxysilane (MTES, Innochem (Beijing, China), AR 98%), acetic acid (CH_3_COOH, Innochem, AR 99.5%). CaSi_2_ powders needed to be purified [18]. We used 2 mol/L NaOH solution to wash CaSi_2_ (Na:Ca = 10:1) for 24 h, then washed with absolute ethanol and dried in a vacuum oven at 60 °C.

### 3.2. Preparation of Siloxene Nanosheets

The SiNSs were prepared referring to previous reports [32]. FeCl_3_ was added to ethyl acetate, a trace amount of water was added, and the dispersion was uniform. The purified CaSi_2_ powders were added and stirred at room temperature for 1 day. The obtained products were centrifuged, and then added to HCl solution (2 mol·L^−1^, 30 mL). The centrifuged precipitate was acid washed for 4 hours to remove the unreacted CaSi_2_. Then, the samples were rinsed with ethanol and centrifuged 3 times. SiNSs were dispersed in anhydrous ethanol and exfoliated by ultrasound. After exfoliation, the samples were centrifuged at low speed and then the upper mixture was collected for nonlinear optical properties testing or dried in a vacuum oven at 50 °C for other powder characterization.

### 3.3. Preparation of Siloxene Nanosheets Hybrid Gel Glasses

The SiNS-glasses were prepared by the sol-gel method [34,39,40]. These gel glasses were prepared by hydrolysis and polycondensation of MTES under acidic conditions (pH = 2.5). We configured solutions A and B separately. The molar ratio of MTES/anhydrous ethanol in solution A is 1:6. The molar ratio of pure water/anhydrous ethanol/glacial acetic acid in solution B is 3.5:6:0.042 (where the same amount of anhydrous ethanol is used in solutions A and B). Solution B was added slowly into solution A under stirring. The mixed solutions were stirred for 1 day, and then half volume of the solvents was evaporated. Subsequently, MTES sols were pre-polymerized by continuous stirring for 1 week. Different concentrations of SiNSs ethanol dispersions were added to the MTES sols, and dispersed uniformly in the sols. The SiNSs hybrid gel was added to the polypropylene molds. Finally, SiNS-glasses were obtained after drying and demolding.

### 3.4. Characterization Method

The morphologies of CaSi_2_ powders were tested by Hitachi S-4800 scanning electron field emission microscope, and samples were dried, glued to the conductive adhesive of the sample table, sprayed with gold, and then tested. The elemental content and distribution of CaSi_2_ was obtained by SEM energy dispersive spectroscopy. The morphologies of SiNSs were tested by Hitachi HT7700 transmission electron microscope (accelerating voltage of 40~120 kV), and the samples were diluted and dropped on the ultrathin carbon film before testing, then observed after drying. The height of SiNSs was measured by atomic force microscope Dimension Fastscan Bio (Bruker, Heidelberg, Germany). The mapping of SiNSs was tested by high-resolution transmission electron microscope JEM-2100F (accelerating voltage of 200 kV), and sample handling was the same as for morphologies testing. XRD pattern was obtained by focusing on D8 (Bruker, Germany). The microstructures of SiNSs were characterized by D8 focus X-ray diffractometer and inVia-Reflex micro confocal Raman spectrometer. The Fourier transform infrared spectra were profiled on an Excalibur HE 3100 (Varian). The elemental compositions of SiNSs present were characterized by ESCALAB 250Xi X-ray photoelectron spectrometer. Transmittance of SiNSs dispersion and UV-Vis absorption spectra were tested with a U-3000 and a Cary 7000 Separately.

The nonlinear optical and optical limiting properties of the SiNSs dispersions and their gel glasses were measured by using open aperture Z-scan apparatus at wavelengths of 532 nm and 1064 nm. The pulse width of the Gaussian laser at 532 nm and 1064 nm was 4.5 ns and 6.5 ns, respectively, from a Q-switched Nd: YAG laser at a repetition rate of 10 Hz. The laser beam was tightly focused with a 20 cm focus lens, and all samples were tested in 1 × 10 mm quartz cuvettes. The beam waist radii at the focus were estimated to be ~13.6 μm and ~27.1 μm for 532 nm and 1064 nm laser pulses, respectively.

## 4. Conclusions

In summary, we reported a top-down topological transformation method to prepare dispersible SiNSs by oxidation and decalcification. The SiNSs structure was intact and the thickness was about 1.5 nm. The siloxene nanosheets were characterized by morphological, structural, and elemental characterization as Kautsky type siloxene nanosheets. We successfully introduced SiNSs into the gel glass using the sol-gel method. The surface groups of the SiNSs enabled the co-polymerization of the nanosheets and the gel glass, resulting in excellent homogeneity of the hybrid gel glass and high linear transmittance in the visible-NIR region. Outstanding nonlinear optical properties were discovered for SiNSs due to low F_ON_. SiNSs hybrid gel glasses have broad-band optical limiting properties that are tuned with changes in hybrid concentration. Consequently, SiNSs and their hybrid gel glasses are promising nonlinear optical material and devices which can be used for optical limiting.

## Figures and Tables

**Table 1:** Element content of CaSi_2_ powders and siloxene nanosheets.

Element	CaSi_2_ Powders		Element	Siloxene Nanosheets	
	Weight%	Atomic%		Weight%	Atomic%
Si K	72.24	78.78	Si K	48.76	35.25
Ca K	27.76	21.22	Ca K	0.36	0.18
			O K	50.88	64.57
Total	100.00	100.00	Total	100.00	100.00

**Table 2:** XPS full spectrum element of siloxene nanosheets.

Element	Weight%	Atomic%
O 1s	40.41	48.37
Si 2p	48.54	33.1
C 1s	11.05	17.63
Totals	100.00	100.00

**Table 3:** F_ON_, R_OL,_ and T_linear_ of the various siloxene nanosheet hybrid gel glasses.

Samples Concentration	532 nm			1064 nm		
(wt%)	F_ON_ (J/cm^2^)	R_OL_ (out/in)	T_linear_ (out/in)	F_ON_ (J/cm^2^)	R_OL_ (out/in)	T_linear_ (out/in)
0.30	0.45	0.15	0.54	1.45	0.32	0.83
0.10	1.60	0.42	0.77	3.32	0.48	0.90
0.03	2.38	0.55	0.91	3.19	0.57	0.93
0.01	2.70	0.62	0.94	4.75	0.70	0.94

**Table 4:** Comparison of the F_ON_ of different materials.

Material	Substrate	Laser Parameters	F_ON_ (J/cm^2^)	Literature
TiS_2_ nanosheets	PMMA glasses	532 nm 7 ns	0.067	[35]
Sb nanosheets	isopropyl alcohol	532 nm 1.8 ns	0.162	[36]
Graphene	NMP	532 nm 6 ns	0.44	[37]
		1064 nm 6 ns	0.64	
Graphene-Ormosil	gel glasses	532 nm 8 ns	0.03	[38]
SiNSs	ethanol	532 nm 4.5 ns	0.28	This work
		1064 nm 6.5 ns	0.53	
	gel glasses	532 nm 4.5 ns	0.45	
		1064 nm 6.5 ns	1.45	

## Data Availability

Not applicable.

## Sample Availability

Samples of the siloxene nanosheets are available from the authors.

## References

[1] Di Piazza A., Mueller C., Hatsagortsyan K.Z., Keitel C.H. (2012). Extremely high-intensity laser interactions with fundamental quantum systems. Rev. Mod. Phys..

[2] Chen Y., Bai T., Dong N., Fan F., Zhang S., Zhuang X., Sun J., Zhang B., Zhang X., Wang J. (2016). Graphene and its derivatives for laser protection. Prog. Mater. Sci..

[3] Zhou G.J., Wong W.Y. (2011). Organometallic acetylides of PtII, AuI and HgII as new generation optical power limiting materials. Chem. Soc. Rev..

[4] Tang Q., Zhou Z. (2013). Graphene-analogous low-dimensional materials. Prog. Mater. Sci..

[5] Xu L., Sun J., Tang T., Zhang H., Sun M., Zhang J., Li J., Huang B., Wang Z., Xie Z. (2021). Metallated Graphynes as a New Class of Photofunctional 2D Organometallic Nanosheets. Angew. Chem..

[6] Jose D., Datta A. (2014). Structures and Chemical Properties of Silicene: Unlike Graphene. Acc. Chem. Res..

[7] Kara A., Enriquez H., Seitsonen A.P., Lew Yan Voon L.C., Vizzini S., Aufray B., Oughaddou H. (2012). A review on silicene—New candidate for electronics. Surf. Sci. Rep..

[8] Guzmán-Verri G.G., Voon L.C.L.Y. (2007). Electronic structure of silicon-based nanostructures. Phys. Rev. B.

[9] Cahangirov S., Topsakal M., Akturk E., Sahin H., Ciraci S. (2009). Two- and one-dimensional honeycomb structures of silicon and germanium. Phys. Rev. Lett..

[10] Houssa M., Pourtois G., Afanas’ev V.V., Stesmans A. (2010). Can silicon behave like graphene? A first-principles study. Appl. Phys. Lett..

[11] Liu J., Yang Y., Lyu P., Nachtigall P., Xu Y. (2018). Few-Layer Silicene Nanosheets with Superior Lithium-Storage Properties. Adv. Mater..

[12] Zhao J., Liu H., Yu Z., Quhe R., Zhou S., Wang Y., Liu C., Zhong H., Han N., Lu J. (2016). Rise of Silicene: A Competitive 2D Material. Prog. Mater. Sci..

[13] Borlido P., Rödl C., Marqes M.A.L., Botti S. (2018). The ground state of two-dimensional silicon. 2D Mater..

[14] Grazianetti C., De Rosa S., Martella C., Targa P., Codegoni D., Gori P., Pulci O., Molle A., Lupi S. (2018). Optical Conductivity of Two-Dimensional Silicon: Evidence of Dirac Electrodynamics. Nano Lett..

[15] Yamanaka S., Matsu-ura H., Ishikawa M. (1996). New deintercalation rection of calcium from calcium disilicide. Synthesis of layered polysilane. Mater. Res. Bull..

[16] Dettlaff-Weglikowska U., Hönle W., Molassioti-Dohms A., Finkbeiner S., Weber J. (1997). Structure and optical properties of the planar silicon compounds polysilane and Wöhler siloxene. Phys. Rev. B.

[17] Nakano H., Ishii M., Nakamura H. (2005). Preparation and structure of novel siloxene nanosheets. Chem. Commun..

[18] Fu R., Zhang K., Zaccaria R., Huang H., Xia Y., Liu Z. (2017). Two-dimensional silicon suboxides nanostructures with Si nanodomains confined in amorphous SiO2 derived from siloxene as high performance anode for Li-ion batteries. Nano Energy.

[19] Krishnamoorthy K., Pazhamalai P., Kim S. (2018). Two-dimensional siloxene nanosheets: Novel high-performance supercapacitor electrode materials. Energy Environ. Sci..

[20] Xu K., Ben L., Li H., Huang X. (2015). Silicon-based nanosheets synthesized by a topochemical reaction for use as anodes for lithium ion batteries. Nano Res..

[21] Pazhamalai P., Krishnamoorthy K., Sahoo S., Mariappan V.K., Kim S.J. (2019). Understanding the Thermal Treatment Effect of Two-Dimensional Siloxene Sheets and the Origin of Superior Electrochemical Energy Storage Performances. ACS Appl. Mater. Interfaces.

[22] Zou J., Baldwin R.K., Pettigrew K.A., Kauzlarich S.M. (2004). Solution Synthesis of Ultrastable Luminescent Siloxane-Coated Silicon Nanoparticles. Nano Lett..

[23] Guo Q., Liu J., Bai C., Chen N., Qu L. (2021). 2D Silicene Nanosheets for High-Performance Zinc-Ion Hybrid Capacitor Application. ACS Nano.

[24] Guo Q., Bai C., Gao C., Chen N., Qu L. (2022). Two Dimensional Silicene Nanosheets: A New Choice of Electrode Material for High-Performance Supercapacitor. ACS Appl. Mater. Interfaces.

[25] You J.W., Bongu S.R., Bao Q., Panoiu N.C. (2018). Nonlinear optical properties and applications of 2D materials: Theoretical and experimental aspects. Nanophotonics.

[26] Papadakis I., Bouza Z., Couris S., Bourlinos A.B., Mouselimis V., Kouloumpis A., Gournis D., Bakandritsos A., Ugolotti J., Zboril R. (2017). Hydrogenated Fluorographene: A 2D Counterpart of Graphane with Enhanced Nonlinear Optical Properties. J. Phys. Chem. C.

[27] Agrawal A., Yi G.C. (2020). Database on the nonlinear optical properties of graphene based materials. Data Brief.

[28] Stavrou M., Papadakis I., Stathis A., Kloberg M.J., Mock J., Kratky T., Gunther S., Rieger B., Becherer M., Lyuleeva-Husemann A. (2021). Silicon Nanosheets versus Graphene Nanosheets: A Comparison of Their Nonlinear Optical Response. J. Phys. Chem. Lett..

[29] Stavrou M., Stathis A., Papadakis I., Lyuleeva-Husemann A., Koudoumas E., Couris S. (2021). Silicon Nanosheets: An Emerging 2D Photonic Material with a Large Transient Nonlinear Optical Response beyond Graphene. Nanomaterials.

[30] Stathis A., Stavrou M., Papadakis I., Mock J., Kloberg M.J., Becherer M., Lyuleeva-Husemann A., Couris S. (2021). Silicon Nanosheets: A Promising 2D Material with Strong Ultrafast Nonlinear Optical Response. J. Phys. Chem. C.

[31] Zhang F., Wang M., Wang Z., Han K., Liu X., Xu X. (2018). Nonlinear absorption properties of silicene nanosheets. Nanotechnology.

[32] Xu X., Zhou L., Ding D., Wang Y., Huang J., He H., Ye Z. (2019). Silicene Quantum Dots Confined in Few-Layer Siloxene Nanosheets for Blue-Light-Emitting Diodes. ACS Appl. Nano Mater..

[33] Meier C., Lüttjohann S., Kravets G., Nienhaus H., Lorke A., Wiggers H. (2006). Raman properties of silicon nanoparticles. Phys. E Low Dimens. Syst. Nanostruct..

[34] Dong X., Wang Y., Guan R., Ren J., Xie Z. (2021). Silane-Functionalized Carbon Dots and Their Polymerized Hybrids: From Optoelectronics to Biotherapy. Small.

[35] Liu Y., Li X., Wang E., Zhong Q., Zhou T., Chen H., Chen S., Lu G., Liang C., Peng X. (2021). Exceptional size-dependent property of TiS2 nanosheets for optical limiting. Appl. Surf. Sci..

[36] Wu J., Wei Y., Shen W., Xiong Y., Lin C., Gao Y., Al-Ammari A., Liu K., Ma T., Chen J. (2020). Antimonene nanosheets fabricated by laser irradiation technique with outstanding nonlinear absorption responses. Appl. Phys. Lett..

[37] Dong N., Li Y., Feng Y., Zhang S., Zhang X., Chang C., Fan J., Zhang L., Wang J. (2015). Optical Limiting and Theoretical Modelling of Layered Transition Metal Dichalcogenide Nanosheets. Sci. Rep..

[38] Zheng X., Feng M., Zhan H. (2013). Giant optical limiting effect in Ormosil gel glasses doped with graphene oxide materials. J. Mater. Chem. C.

[39] Xie Z., Wang F., Liu C. (2012). Organic-inorganic hybrid functional carbon dot gel glasses. Adv. Mater..

[40] Xing F., Wang J., Wang Z., Li Y., Gou X., Zhang H., Zhou S., Zhao J., Xie Z. (2021). Covalently Silane-Functionalized Antimonene Nanosheets and Their Copolymerized Gel Glasses for Broadband Vis-NIR Optical Limiting. ACS Appl. Mater. Interfaces.

